# A model to support radiation therapists in facilitating sexual health counselling for patients with prostate cancer

**DOI:** 10.4102/hsag.v30i0.3047

**Published:** 2025-10-17

**Authors:** Nape M. Phahlamohlaka, Penelope Engel-Hills, Hesta Friedrich-Nel

**Affiliations:** 1 Department of Medical Imaging and Therapeutic Sciences, Faculty of Health and Wellness Sciences, Cape Peninsula University of Technology, Cape Town, South Africa; 2 Department of Clinical Sciences, Faculty of Health and Environmental Sciences, Central University of Technology, Free State, Bloemfontein, South Africa

**Keywords:** supportive care, oncology health professional, radiotherapy, sexual health, counselling, radiation therapy, model framework

## Abstract

**Background:**

The involvement of radiation therapists (RTTs) in providing supportive care for patients facing sexual health challenges during radiotherapy for prostate cancer remains inconsistent in radiation therapy settings and under-researched in the South African context.

**Aim:**

The aim was to develop a model framework to support the facilitation of men’s sexual health for patients with prostate cancer by leveraging the role of RTTs.

**Setting:**

This study was conducted in the radiation therapy departments of two academic hospitals in Gauteng, South Africa.

**Methods:**

A theory-generating design consisting of four steps was used to develop the model framework: (1) identifying, defining and classifying the central concepts, (2) constructing relationship statements, (3) describing the model and (4) evaluating the model.

**Results:**

The central concept drawn from the empirical findings is the *facilitation of supportive care for men’s sexual health*. A model was developed, described and evaluated.

**Conclusion:**

The model framework has the potential to improve the inclusion of conversations by RTTs on sexual health as part of routine care for patients receiving radiotherapy for prostate cancer.

**Contribution:**

This article presents a model to promote patient counselling practices for RTTs that involve supportive care for men’s sexual health.

## Introduction

Supportive care for patients treated for prostate cancer with sexual health challenges in radiation therapy settings is challenging for oncology health professionals, including radiation therapists (RTTs), and is often an overlooked or avoided aspect of care (Bingham, Cassells & Semple 2024; Krouwel et al. 2019; Oskay, Can & Basgol 2014; Phahlamohlaka & Mdletshe 2022). Although oncology practitioners recognise that discussing sexual health falls within their professional responsibilities, such conversations are not consistently integrated into routine practice because of systemic challenges, such as lack of time and shortage of staff (Bräutigam et al. 2020; Krouwel et al. 2019). This practice gap in supportive care for patients with cancer highlights the need to expand its focus beyond oncology practitioners and engage the broader oncology team of health professionals to include RTTs.

Radiation therapists are expected to advise patients with cancer on radiotherapy side effects and offer psychosocial counselling in collaboration with radiation oncologists (Australian Society of Medical Imaging and Radiation Therapy 2015; Health Professions Council of South Africa 2021). Moreover, RTTs are best known for maintaining regular therapeutic interactions with patients with cancer throughout radiotherapy treatment (Egestad 2013; Mattarozzi et al. 2019). Several studies have indicated that RTTs are well positioned to assist with the provision of emotional support, and they can discuss sensitive topics, such as sexual function and intimacy, with patients receiving radiotherapy for cancer (Flood et al. 2023; Hulley et al. 2016; Lynch, O’Donovan & Murphy 2019; Mattarozzi et al. 2019; Van Beusekom et al. 2019). Although the involvement of RTTs in facilitating sexual health conversations with patients is under-researched, there are limited emerging and convincing studies to support the role of RTTs in providing supportive care during radiotherapy for patients experiencing sexual health challenges (Martino & Odle 2007; Nisbet, Caulfield & Holloway 2021; Oliveira et al. 2022; Turner 2019; Turner & Di Prospero 2015).

Several sexual health communication frameworks exist in the literature to guide healthcare professionals in initiating sexual health conversations across healthcare clinical settings. These frameworks include Extended Permission, Limited Information, Specific Suggestion, Intensive Therapy (EX-PLISSIT) and Bringing Up, Explaining, Telling, Timing, Education, Recording (BETTER) and Engagement, Assessment, Support, Sign-posting (EASSI). EX-PLISSIT and BETTER provide structured approaches to discussing sexual concerns (McCaughan et al. 2020; Quinn, Happell & Welch 2012). Nevertheless, it is posited that current frameworks are generalised, and without the provision of a clear delineation of professional roles or sufficient contextual specificity regarding the clinical environments and health professionals, they are designed to support. Additionally, a theory-driven framework tailored to underpin the support for the practice of RTTs’ involvement in facilitating sexual health counselling with patients receiving radiotherapy for prostate cancer could not be located at the time of the study in the literature.

This article aims to present a contextually grounded model framework developed to leverage the role of RTTs to enhance supportive care for men’s sexual health (SCMSH) during radiotherapy for prostate cancer. This model is expected to address existing gaps in supportive care for patients experiencing sexual challenges during radiotherapy for prostate cancer, thereby improving the delivery of whole-person care in oncology settings.

## Research methods and design

### Study design

The study, which underpins the model described in this article, employed a sequential exploratory multi-method research design for data collection (Creswell & Creswell 2018; Vivek & Nanthagopan 2021). This design was selected to explore patient experiences regarding sexual health support in oncology settings and to survey the perspectives of RTTs regarding their involvement in facilitating such support for patients receiving radiotherapy for prostate cancer.

### Population and sampling

The target populations represented two participant cohorts. Cohort 1 comprised all patients who had received radiotherapy for prostate cancer in the past 6 months or longer as of January 2021. Cohort 2 consisted of a population of 60 RTTs. In this study, 12 patients from Cohort 1 participated in interviews, after which data saturation was deemed to have been reached. A sample of 50 respondents was calculated for Cohort 2. Participants in Cohort 1 were purposively selected based on specific characteristics to meet the objective of the study (Benoot, Hannes & Bilsen 2016; Campbell et al. 2020). Cohort 2 employed convenience sampling, selecting participants not only based on the inclusion criteria but also based on their availability and accessibility. This method was appropriate considering practical constraints, including time limitations and limited access to radiation therapy departments in South Africa (Etikan, Musa & Alkassim 2016). The research setting for the study was the therapy departments at two public hospitals in Johannesburg and Pretoria in Gauteng, South Africa.

### Data management

Participants were provided with an information sheet describing the research aim and indications for participation in the study. All participants were notified about their voluntary participation and right to withdraw from the study at any time without providing a reason and with no negative consequences. All data generated from this study were de-identified and electronically stored on Microsoft SharePoint, accessible to the researchers through a password. The first author obtained written informed consent from the male patients in Cohort 1 before the commencement of interviews. Implicit consent was obtained for the survey to protect the anonymity of the RTTs in Cohort 2. The first page of the questionnaire included a statement that completing and returning the questionnaire constituted implied consent to participate in the study (Manandhar & Joshi 2020). The narrative responses were captured and stored in the first author’s password-protected QuestionPro online survey software account. Completed paper-based questionnaires will be destroyed after research articles related to this study have been published. Lastly, a panel of experts completed the online express consent to participate in the model evaluation.

### Research instruments

Data were collected through face-to-face interviews with patients and questionnaires administered to RTTs. A pilot study was conducted with two patients to assess the interview setting, test the audio recording device and check whether the questions in the interview guide were clear. The development of the questionnaire was carried out with guidance from findings from Cohort 1. The questionnaire was pilot tested with five RTTs to check its content validity.

### Data collection procedure

The timeframe for data collection spanned from 01 January 2021 to 30 November 2021, including a pilot study phase for both cohorts. Cohort 1 research was conducted before Cohort 2. In Cohort 1, the first author scheduled an appointment with the patients for a face-to-face interview after recruiting participants in the selected hospital during clinic follow-up reviews. All participants were asked two key questions: ‘How did you experience the support provided on sexual health during and after radiotherapy in the oncology clinic?’ and ‘What advice did you receive from *radiation therapists* regarding sexual health during your radiotherapy treatment?’ These were followed by probing questions based on their responses. The interviews lasted approximately 45 min each. In Cohort 2, a questionnaire with open-ended questions was developed to explore RTTs’ perspectives on the inclusion of sexual health support into routine patient counselling for patients receiving radiotherapy for prostate cancer. A research assistant distributed questionnaires to RTTs at research sites; a drop box was placed at each research site to collect completed questionnaires. The drop boxes were removed from the hospitals after 30 days, after a verbal notice was given a week before by a research assistant.

### The model development process

**FIGURE 1:**
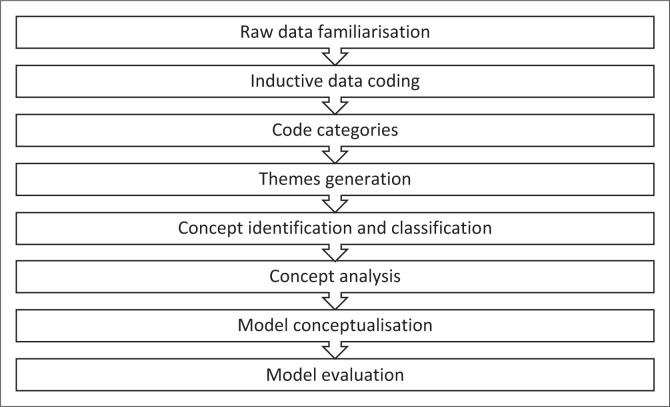
Conceptual framework for the model development process.

The development of a model framework followed four steps: (1) identifying, defining and classifying the central concepts, (2) constructing relationship statements, (3) developing the model and (4) evaluating the model (Foley & Davis 2017; Walker & Avant 2019). Concept identification was carried out following Naeem’s inductive thematic data analysis of the interview data of participants and open-ended textual responses of survey respondents for model conceptualisation (Brownstone et al. 2021; Naeem et al. 202) and Dickoff’s theory for classifying identified concepts from the findings (Dickoff, James & Wiedenbach 1968). The analysis of concepts adhered to the methodology outlined by Walker and Avant (2019), which culminated in conceptualising the central concept of the model framework. Model evaluation was conducted using the criteria of clarity, simplicity, transferability, accessibility and importance, with the input from a panel of experts (Chinn & Kramer 2015). The conceptual framework guiding the development of the model presented later in this article is illustrated in Figure 1.

### Trustworthiness

The trustworthiness of the overall study was assessed using Lincoln and Guba’s (1985) four criteria for trustworthiness: credibility, transferability, dependability and confirmability (Morse et al. 2002; Shenton 2004). Truth value was ensured through prolonged participant engagement and expert review of the model, supported by the researcher’s subject knowledge. Detailed context descriptions and transparent methodology enhanced transferability. Dependability of the study was achieved through the independent transcription of interviews and the use of patient interviews and RTT questionnaires for data collection to develop multiple perspectives on the phenomenon being studied. Dependability was also ensured by providing an audit trail detailing the processes leading to model development (Holloway & Galvin 2023). Confirmability confirms that the researcher’s preconceptions have not influenced the findings and conclusions of the study (Fouché, Strydom & Roestenburg 2021; Kumar 2014). Confirmability was ensured through reflexivity and bracketing, with the first author setting aside personal biases. Theme verification was carried out in consultation with the study leaders. To ensure that the survey instrument was valid, it was piloted with five RTTs, ensuring that the instrument accurately captured the construct it intended to explore.

### Ethical considerations

The principles of autonomy, non-maleficence and beneficence were upheld in the conduct of the research study (Dhai & MacQuoid-Mason 2011; Varkey 2021). Ethical clearance was obtained from the Cape Peninsula University of Technology (CPUT/HW-REC 2020/H15) and the University of the Witwatersrand (M2011123). Gatekeeper permission was obtained from the heads of the oncology departments. This study was registered with the National Health Research Database of South Africa (GP_202011_082).

## Results and discussion

An exploration of patient experiences and RTT perspectives reveals gaps in the facilitation of SCMSH in oncology settings for men receiving radiotherapy for prostate cancer. Patients have consistently reported ongoing sexual health challenges, with limited communication about these challenges with oncology health professionals. The RTTs acknowledged the significance of addressing these issues but indicated that they did not regularly engage in such conversations. Following the four theory-generative steps described by Chinn and Kramer (2015), this article presents a model framework intended to promote the involvement of RTTs in promoting men’s sexual health for patients with prostate cancer.

### Step 1: Identifying, defining and classifying the central concepts

**FIGURE 2:**
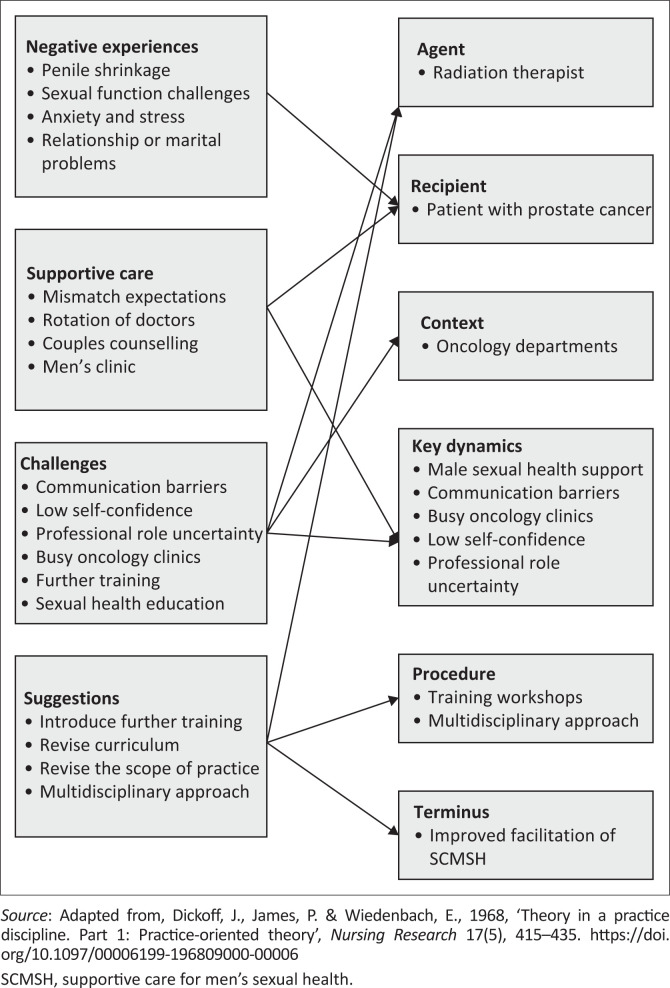
Classification of concepts according to Dickoff’s theory.

Key concepts identified include erectile dysfunction, intimacy, supportive care, mismatched expectations, sexual health, men’s clinic, communication barriers, time constraints, self-confidence, need for further training, scope of practice and multidisciplinary care. A broad classification of these concepts according to Dickoff’s theory (Dickoff et al. 1968) is shown in Figure 2.

The key concepts identified were classified using the practice theory of Dickoff, as shown in Figure 2 (Chinn & Kramer 2015; Dickoff et al. 1968). The six elements of this theory, with examples specific to the supportive care model, are the: (1) recipient (male patient), (2) agent (RTT), (3) context (oncology), (4) dynamics (men’s sexual health challenges, communication barriers), (5) procedure (further training and multidisciplinary approach) and (6) terminus (improved facilitation of SCMSH) (Dickoff et al. 1968).

### Step 2: Constructing the relationship statements

The identified and classified concepts were written into relationship statements to establish a logical understanding of the connection between the concepts in terms of agent, recipient, context, dynamics, procedure and terminus (Chinn & Kramer 2015; Dickoff et al. 1968). The relationship statements defined for the model framework in this article are as follows:

- When RTTs are given targeted training and institutional support on sexual health within oncology departments, they are more likely to engage confidently and effectively in sexual health conversations with patients with prostate cancer to address their challenges.
- Negative experiences of sexual health challenges, relationship strain and unmet expectations among patients with prostate cancer underscore the need for RTTs to play an active role in providing supportive sexual health counselling during radiotherapy.
- Persistent barriers such as limited communication skills, low self-confidence and uncertainty regarding the scope of practice among RTTs hinder the effective facilitation of sexual health support in oncology settings.
- In radiation therapy departments characterised by high clinical workloads, patients may receive inconsistent or inadequate sexual health counselling, warranting RTTs to play an active role in sexual health counselling with patients treated for prostate cancer.
- Aligning training content and scope-of-practice expectations with the practical realities of oncology care can enable RTTs to contribute meaningfully to improving the supportive care of men’s sexual health with patients with prostate cancer.

### Step 3: Describing the model framework

**FIGURE 3:**
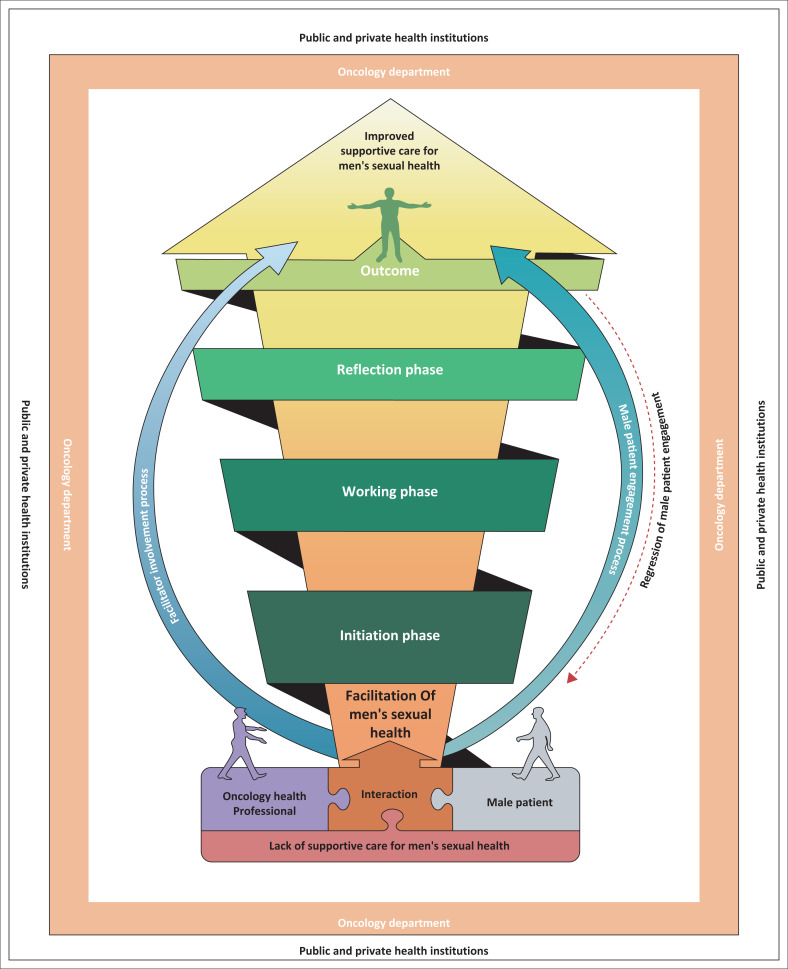
Proposed model to support the involvement of radiation therapists in facilitating supportive care for men’s sexual health.

The proposed model to facilitate SCMSH, depicted in Figure 3, is described in terms of its purpose and context, definition of concepts, structure and nature and model process, following the guidelines of Chinn and Kramer (2015).

#### Purpose and context

The model is designed to facilitate SCMSH in patients receiving radiotherapy for prostate cancer, specifically within the context of South African oncology settings. The oncology context in this article refers to the radiation therapy department.

#### Definition of the central concept

The central concept synthesised for this model is the *facilitation of supportive care for male sexual health*. The sub-concepts *facilitation, supportive care* and *male sexual health* were defined using scholarly sources and reputable online dictionaries (Collins Dictionary 2024; Cramp & Bennett 2013; Henwood & Taket 2008; Merriam-Webster Dictionary 2025a; World Health Organization [WHO] 2006). The operational definition of the central concept is the delivery of comprehensive support to patients receiving radiotherapy for prostate cancer by addressing their informational, emotional, spiritual, social and physical needs, fostering whole-person care throughout radiotherapy.

**Facilitation:** The Collins Dictionary (2024) defines *facilitation* as an action or process, especially one that an individual would like to happen, which means making it easier or more likely to occur. The spectrum of facilitation is broad, ranging from creating a space in which everybody can have their say to facilitating processes where people can:

‘Face and take on board the unknown, through letting go of old knowledge, habits or ways of seeing … to step over existing boundaries, entering and transcending the threshold of change.’ (Soal 2004:55).

Supportive care: The concept of *supportive* involves actively helping someone in need of help, whereas *care* means paying serious attention, especially to the details of a situation or thing (Cambridge Dictionary 2025a). The Merriam-Webster Merriam-Webster Dictionary 2025a defines *care* as a feeling of interest or concern. In the study context, *supportive care* is the provision of necessary support to those living with or affected by cancer to meet their informational, emotional, spiritual, social and physical needs during their diagnosis, treatment and follow-up phases (Hui 2014).

Men’s sexual health: To define men’s sexual health, the concept was divided into three sub-concepts: *men, sexual* and *health*. In the Cambridge Dictionary (2025b), *men* are defined as those belonging to or related to the male gender. *Man* is an individual human being, particularly an adult male Merriam-Webster Dictionary 2025b. The adjective *sexual* is related to the instincts, physiological processes and activities connected with physical attraction or intimate physical contact between individuals (Bab.la 2025). *Health* refers to well-being that encompasses a person’s physical, mental and social aspects with specific reference to reproductive and sexual functions, soundness of the body or mind and freedom from disease or ailments Dictionary.com 2023; Merriam-Webster Dictionary 2025c.

#### The structure and nature of the model

The outer white frame with square borders in Figure 3 represents the context of public and private healthcare facilities. The inner frame represents the oncology department within the public and private institutions where men’s sexual health support should be offered. The red puzzle piece at the bottom of the model, connected to the orange interconnecting puzzle piece, represents the problem that this model seeks to address. The violet figurine on the bottom left side represents the facilitator (agent), whereas the grey figurine on the right side represents the male patient (recipient). The dark orange centre puzzle piece at the bottom depicts an opportunity for two-way communication on men’s sexual health issues between the oncology health professional or RTT (left) and the male patient (right).

On the left side of the large central arrow is a blue upward-curved arrow with a broad base that gradually becomes thinner towards the end. The tapering nature of the blue arrow and the transition from dark to light blue towards the arrowhead represent the intensity of facilitator involvement throughout the model process. On the right side of the large central arrow is a narrow blue-green (teal) arrow that curves upward with a broadened endpoint, symbolising the improved experience of male patients regarding SCMSH. The intensity of blue-green changes as the curve widens from the base to the arrowhead. This indicates the extent to which the male patient is engaged in and responsive to SCMSH facilitated by an oncology health professional. As the patient receives sexual health support, the widening arrow suggests that the patient develops stability in the internal environment during the initiation, working and reflection phases.

The central upward orange-yellow arrow gradually widens to reflect a positive change in the facilitation of SCMSH in oncology. In the context of this model, the medium orange colour used symbolises a warm, conducive space for male patients to interact with an oncology health professional and freely share their feelings about their experience of living with erectile dysfunction (McLeod 2016). The widened arrowhead indicates the improved SCMSH as a result of the model process.

#### Model process

The SCMSH facilitation process has three phases: initiation, working and reflection. The green zigzag loop, intertwining the large upward-pointing, widening orange-to-yellow arrow, illustrates these phases. Green represents physical, emotional and mental rejuvenation during episodes of distress about erectile dysfunction (Cherry 2023; Learn Laugh Speak 2022). The intensity of the green colour differs to represent the level of engagement of the oncology health professional with the male patient.

**Initiation phase:** The oncology health professional in this context is an RTT approaching the patient with open arms and extending his or her hands towards the patient, showing confidence and commitment to offering SCMSH to a patient with prostate cancer. This implies that the oncology health professional should initiate dialogue with the patient rather than the patient having to take the lead. The facilitator’s gender is irrelevant, even though the violet figurine representing the oncology health professional shown in Figure 3 indicates a male person. In this phase, the facilitator develops a therapeutic bond with the patient by establishing a safe, trusting and non-judgemental environment for the patient to speak freely. This phase is the largest of the three because the facilitator needs ample time to build a therapeutic relationship with the patient. The dark green hue representing this phase indicates the effort and energy (intensity) the facilitator requires to initiate SCMSH with new patients. During this phase, the facilitator and patient set actionable goals.

The grey figurine, portraying a male patient seeking supportive care for his sexual health concerns from the oncology health professional, symbolises someone experiencing low morale, stress, frustration and hopelessness. The facilitator’s level of involvement is indicated by the blue arrow, which has a broad base during the initiation phase. The curved arrow gradually becomes narrower as it passes through the working and reflection phases, thereby representing a decrease in the level of facilitator involvement. The facilitator’s engagement level is high in the initiation phase, as the oncology health professional attempts to build a safe, trusting and amicable environment so that the patient can speak freely. The gradual change in the intensity of the blue colour from the initiation phase to the reflection phase throughout the facilitation process explains this.

The engagement process of the male patient is represented by the curved blue-green (teal) arrow with a narrow base in the initiation phase, which becomes progressively thicker in the working and reflection phases. Variation in the hue of the teal colour from light to dark indicates the extent to which the patient engages with the oncology health professional during the facilitation of men’s sexual health. The changing thickness of this arrow represents an increase in the self-confidence and trust of the patient during the process. This change is symbolic of the gradual improvement in men’s positive experiences with supportive care for their sexual health. The facilitator transitions to the working phase only when the patient is prepared to discuss sexual health challenges. If the patient is not ready, the facilitator can offer reassurance with phrases such as, ‘Today may not be the best time to discuss your sexual health challenges with me, but please let me know when you feel comfortable having this conversation.’

**Working phase:** During this phase, the communication between the facilitator and the patient evolves into a two-way conversation. The facilitator must continue to value and respect the patient’s preferences, health beliefs, culture, religion and societal norms because these factors are often barriers to sexual health dialogue that can result in a patient not engaging in the process (Arousell & Carlbom 2016; Degni et al. 2012). The goal of this phase is to help the patient restore balance in their body, mind and spiritual dimensions that have been disrupted by a decline in sexual health and challenges causing intimate-relationship problems. Supportive care for men’s sexual health moves from the initiation phase to the reflection phase with the facilitator utilising the whole-person-care approach. The patient is given time to develop mental strength and cope with external environmental stressors that exert pressure on him to regain erectile function. The progression of supportive care is subtle and marked in the model by the gradual colour change of the spiral loop from dark green to lighter green. In practical terms, this means that the patient starts to achieve balance in the internal environment of their body, mind and spirit to overcome stressors. Lastly, when complex issues arise beyond the facilitator’s expertise while the facilitator is engaging with the patient, it is essential to refer the patient to a specialist, such as a clinical psychologist or sex therapist, for expert advice.

**Reflection phase:** The reflection phase is the last phase and is represented in light green, indicating renewal and optimism (Cherry 2020). In this phase, the oncology health professional and male patient engage in debriefing to reflect on the facilitation of the patient’s sexual health. This final phase is smaller and less intensive than the initiation and working phases. In this phase, the patient reflects on the gaps, successes and strengths of the facilitation process. The oncology health professional and male patient reflect on whether their shared objectives were met and evaluate the successes and shortcomings of the entire model process. Furthermore, the oncology health professional and male patient contemplate interventions that the patient could use to cope with erectile function challenges or rehabilitate sexual functioning to improve the quality of life during and after radiotherapy for prostate cancer. The facilitator’s involvement in this phase is limited and is mainly geared towards the patient, who reflects on his satisfaction following the facilitation of SCMSH during radiotherapy for prostate cancer. The upper third portion of this model shows the outcome of the initiation, working and reflection phases. The outcome is improved facilitation of SCMSH. The male patient, initially portrayed in grey, as shown in Figure 3, is now transformed into green. This figurine represents a happy man with enhanced self-confidence who is content with himself and ready to deal with external environmental factors that may destabilise his internal environment. The assumption here is that, at this stage, the patient will have worked out a way to cope with his sexual health challenges. Theoretically, this means that the male patient has regained a balance in the internal environment of body, mind and spirit to overcome the stressors of the external environment, achieving whole-person care. As the patient starts to restore balance in the body, mind and spirit during the initiation, working and reflection phases, the role of the facilitator gradually decreases, as indicated by the tapering end of the curved arrow and its narrow arrowhead at the top left-hand side of the model. The male patient engagement process curve, culminating in a thick, dark blue-green arrowhead at the top right-hand side, signifies the positive SCMSH experience that the patient received in the oncology department.

The regression of the male patient’s engagement process defines a relapse activity. The dashed red line indicates a scenario in which the patient either did not actively participate or chose to withdraw from the interaction, leading to no positive outcomes. A relapse in the model process indicates that the patient may revert to or persist in an unbalanced internal state, marked by hopelessness, loneliness, reduced confidence and fear of intimate relationships, symbolised by the grey male figurine. The red hue signifies potential flaws or challenges in the facilitation process, which might necessitate termination, repetition or referral to a specialist (Fikrlova et al. 2019) based on the patient’s wishes.

### Step 4: Evaluation of the model

A panel of four experienced experts evaluated the model to facilitate SCMSH. The selected experts had experience in model development, radiography education and practice and nursing education and practice. All panel members had a PhD, and one was a professor. Table 1 shows the demographics of the expert panel members.

**TABLE 1:** Demographics of the expert panel members.

Expert	Qualification	Experience (years)	Designation	Sector	Country
ER 1	PhD in Radiography	30	Senior lecturer	Academia	Australia
ER 2	PhD in Nursing	24	Professor	Academia	South Africa
ER 3	PhD in Radiography	8	Lecturer	Academia	South Africa
ER 4	PhD in Radiography	20	Senior lecturer	Academia	South Africa

ER, expert reviewer; PhD, Doctor of Philosophy.

The evaluation to review and critique the model took place online in a meeting convened via Microsoft Teams. The first draft of the model was shared with the experts by email before the meeting was held. The comments made by the panel experts to enhance the first draft of the model included the following:

- Adjust the size of the grey figurine to match the size of the green figurine.
- Rename the orientation phase to the initiation phase.
- Rename the termination phase to the reflection phase.
- Adjust the spiral model process sizes to reflect activity in each phase.

Following the online review meeting, the panel assessed the model via a Google Forms link using Chinn and Kramer’s criteria (2015), which included clarity, simplicity, transferability, accessibility and importance. The panel concluded that the model concepts are simple, clear, straightforward and easy to follow. The expert panel deemed the model valuable and applicable across private and public oncology settings. Researchers also share the same sentiments based on similarities in professional care at public and private oncology departments. While the model places a strong emphasis on the role of RTTs in improving the facilitation of SCMSH, it is also transferable to other oncology health professionals, as oncology care is inherently a multidisciplinary practice. The panel and researchers agree that the model framework will play a vital role in addressing the existing gap in supportive care for male patients experiencing sexual health challenges during radiotherapy by drawing on the expertise of RTTs in patient care.

## Original contribution

The model put forward in this article is an original contribution to the radiation therapy profession, as to the authors’ knowledge, there is no model framework currently available to recognise and support the involvement of RTTs in facilitating SCMSH for patients on treatment for prostate cancer in radiation therapy settings. This model recognises and promotes the involvement of RTTs in facilitating SCMSH among patients with cancer, aiming to strengthen such care within the unique context of the South African healthcare system.

### Limitations

The scope of this study was limited to two key population groups: men who had completed radiotherapy for prostate cancer and RTTs from two radiation therapy departments in public hospitals in the Gauteng province. The proposed model framework has not been tested in a real-world oncology setting. Nevertheless, feedback from the expert panel suggests that the model holds considerable potential and is regarded as feasible for implementation in oncology settings to strengthen SCMSH through active involvement of RTTs.

### Recommendations

The researchers suggest improved collaboration among oncology health professionals, including but not limited to RTTs, to create a conducive environment for better facilitation of SCMSH during radiotherapy for prostate cancer. Universities offering oncology-related studies should integrate sexual health topics into oncology health trainees’ education and training curricula. Lastly, researchers recommend a review of the scope of practice for RTTs to make their role less ambiguous concerning the provision of sexual health counselling to patients on cancer treatment.

## Conclusion

The article presents a reference model framework to lay a foundation for enhancing SCMSH for patients receiving radiotherapy for prostate cancer by leveraging the role of RTTs within South African oncology departments. This model supports and advocates for the involvement of RTTs to facilitate the integration of sexual health support into the routine care of patients receiving radiotherapy for prostate cancer, thus contributing to filling the gaps in SCMSH in this oncology context. Enhancing supportive care for patients with prostate cancer with sexual health challenges requires a multidisciplinary approach that extends beyond the sole responsibility of oncology clinicians. The active engagement of other key oncology health professionals, particularly RTTs, is essential to advance comprehensive patient care. The researchers assert that this model shows promise for being applicable in environments similar to the South African oncology context.
